# Hepatocellular carcinoma cases with high levels of c-Raf-1 expression may benefit from postoperative adjuvant sorafenib after hepatic resection even with high risk of recurrence

**DOI:** 10.18632/oncotarget.3799

**Published:** 2016-03-13

**Authors:** Jianyong Lei, Jinjing Zhong, Jingcheng Hao, Zhengni Liu, Peng Zhang, Lixue Wu, Lunan Yan, Jinqiang Zhu, Yong Zeng, Bo Li, Tianfu Wen, Wentao Wang

**Affiliations:** ^1^ Thyroid and Parathyroid Surgery Center, West China Hospital of Sichuan University, Chengdu, China; ^2^ Liver Surgery, West China Hospital of Sichuan University, Chengdu, China; ^3^ Department of Pathology, West China Hospital of Sichuan University, Chengdu, China; ^4^ Third General Surgery, The First Hospital of Handan, Handan, Hebei, China; ^5^ State Key Laboratory of Biotherapy/Collaborative Innovation Center of Biotherapy, West China Hospital, Sichuan University, Chengdu, China

**Keywords:** hepatocellular carcinoma, recurrence, sorafenib, risk factors, liver resection

## Abstract

**Background and Aims:**

Liver resection combined with postoperative sorafenib to prevent recurrence remains a controversial approach for cases of hepatocellular carcinoma (HCC), especially cases with a high risk of recurrence. This study aimed to investigate the efficacy and safety of liver resection combined with sorafenib for HCC with a high risk of recurrence.

**Results:**

Most of the cases of HCC were caused by hepatitis B virus (HBV) infection (23 cases, 92%). Most of these tumors (21 cases, 84%) were stage III according to the TNM staging system (12 cases with IIIa, 9 cases with IIIb). In the months after hepatic resection, 19 of the 25 cases (76%) were diagnosed with HCC recurrence or metastasis. Based on the tumor histological biomarker grading system, the group with higher expression levels of c-Raf-1 showed significantly longer overall survival than the group with lower expression of c-Raf-1 (*P* = 0.012). However, the long-term tumor-free survival advantage disappeared (*P* = 0.061). Univariate and multivariate analyses indicated that higher expression of c-Raf-1 was significantly associated with better overall survival (hazard ratio [HR]: 1.842; 95% confidence interval [CI]: 1.211–2.542; *P* = 0.031) and tumor-free survival (HR: 1.319; 95% CI: 1.017–1.543; *P* = 0.046) in HCC patients who underwent radical hepatic resection.

**Patients and Methods:**

We retrospectively collected 25 HCC cases with a high risk of recurrence who underwent radical liver resection and who took sorafenib postoperatively from Jan 2010 to Dec 2012. Factors that might contribute to tumor recurrence and treatment failure such as clinical factors, tumor features, and molecular biomarkers were included in our analysis.

**Conclusions:**

HCC patients with a high risk of post-hepatic resection recurrence may benefit from postoperative sorafenib administered as an adjuvant therapy, especially in cases with high levels of c-Raf-1 expression on histological examination.

## INTRODUCTION

Hepatocellular carcinoma (HCC) is the sixth-most common type of cancer worldwide and the third-most common cause of cancer-related death [1]. Currently, local ablation, liver resection and liver transplantation offer the best chances for a cure. However, ablation can only be applied to small targets, and the liver graft shortage also limits the use of LT for HCC [2]. Thus, liver resection may be the best choice for early-stage HCC [3]. However, some studies have suggested that liver resection should also be applied to intermediate HCCs [4] and even in some advanced HCCs [5]. However, HCC recurrence and subsequent death are common [5, 6]. Many protocols have been used to prevent HCC recurrence, including postoperative adjuvant TACE [7, 8], hepatic arterial infusion therapy [9], administration of sorafenib [10], and other methods [11].

Sorafenib, a multikinase inhibitor, is thought to exert its antitumoral effects through the inhibition both angiogenesis and tumor cell proliferation by receptor tyrosine kinases, such as the serine-threonine kinases Raf-1, VEGFR1-3, and PDGFR-β. These agents have shown modest efficacy, resulting in improved survival inpatients with advanced HCC [12–14]. However, their utility in a postoperative adjuvant setting, especially for cases with a high risk of recurrence, is not known [12, 15]. Herein, we investigated the safety and efficacy of post-resection sorafenib for cases with a high risk of recurrence.

## RESULTS

### Baseline characteristics

As shown in Table 1, the baseline characteristics of the 25 HCC patients were compiled; all of the patients were male, most of the HCC cases were caused by HBV infection, and only 2 cases exhibited an absence of hepatitis virus infection, including HCV and no liver cirrhosis. Only 2 cases with liver function were classified as Child-Pugh B, and most cases were Child-Pugh A. The average total diameter was 7.0 cm, and the average largest tumor diameter was 6.2 cm; most of these patients had a solitary tumor diameter (15 cases, 60%), and 4 cases had multiple tumor targets (i.e., more than 3 targets). The AFP levels ranged from 0 to 400 ng/ml in 13 cases, from 400 to 800 ng/ml in 3 cases, and were more than 1210 ng/ml in 9 cases; most of the tumors in these patients (21 cases, 84%) were stage III according to the TNM staging system (12 cases with IIIa, 9 cases with IIIb).

**Table 1:** Baseline demographic and tumor characteristics of the 25 patients

Patient number	25
Age	50.3±10.1
Gender (M/F)	25/0
Height (cm)	170.4±4.3
Weight (kg)	69.5±8.6
BMI (kg/m^2^)	24.8±4.2
Underlying liver disease (HBV/Negative)	23/2
HBV-DNA (+/−)	14/11
Child-Pugh score (A/B)	23/2
Hemoglobin(g/L)	142.3±20.9
Platelet (×10^9^/L)	143.2±62.9
Creatine	81.4±10.2
Total bilirubin	16.3±7.7
ALB (g/L)	40.8±4.9
NLR(<4/≥4)	21/4
Total tumor size(cm)	7.0±2.6
Largest tumor size (cm)	6.2±2.7
Tumor number (1/2/3/multiple)	15/5/1/4
AFP level (−/+/++/+++)	13/3/0/9
Risk factor (MI/STs/RMLT1/multiple)	10/1/11/3
TNM stage (I/II/III)	1/3/21

M: male; F: female; BMI: body mass index; HBV: hepatitis B virus; HCV: hepatitis C virus; AFP: alpha-fetoprotein; NLR: neutrophil/lymphocyte ratio; ALB: albumin; AFP level: -, 0–400 ng/ml; +, 400–800 ng/ml; ++, 800–1200 ng/ml; +++, ≥1210 ng/ml; MI: microvascular invasion; STs: satellite targets; RMLT1: resection margins less than 1 cm; TNM: tumor node metastasis.

### Tumor recurrence and long-term survival

**Figure 1:**
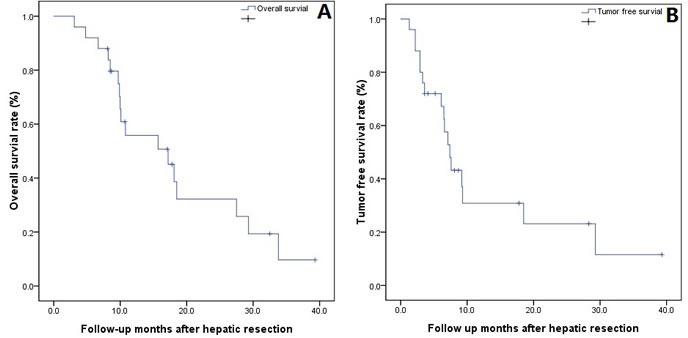
A-B: The overall **A.** and tumor-free survival; **B.** following hepatic resection in 25 HCC patients.

The overall survival and tumor-free survival rates in these 25 cases are shown in Figure 1A and 1B. In the months following hepatic resection, 19 (76%) of the 25 cases were diagnosed with HCC recurrence or metastasis; the most common site of HCC recurrence was the liver (14 cases), followed by liver recurrence and lung metastasis (2 cases), only lung metastasis (1 case), liver recurrence and bone metastasis (1 case), and liver recurrence and abdominal lymph node metastasis (1 case). To date, 17 cases have died, and all of the deaths in these patients were caused by HCC recurrence or metastasis. Additionally, the most common treatments for recurrence or metastasis were TACE (13 cases), TACE combined with RFA (2 cases), re-hepatic resection (2 cases), or TACE combined with LT (1 case).

### Levels of tumor histological biomarkers

**Figure 2:**
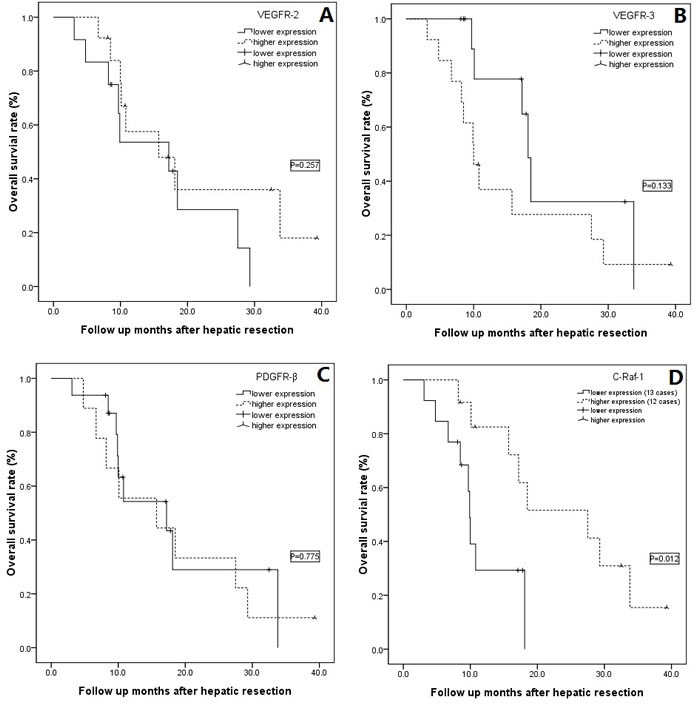
A-D: Subgroup overall survival analysis according to VEGFR-2/3, PDGFR-β and c-Raf-1 expression **A.** No difference was detected in the overall survival between the VEGFR-2 low- and high-expression groups (*P* = 0.257); **B.** No difference was detected in the overall survival between the VEGFR-3 low- and high-expression groups (*P* = 0.133); **C.** No difference was detected in the overall survival between the PDGFR-β low- and high-expression groups (*P* = 0.775); **D.** The cases with high levels of c-Raf-1 expression showed significantly higher overall survival than the cases with lower levels of this biomarker (*P* = 0.012).

**Figure 3:**
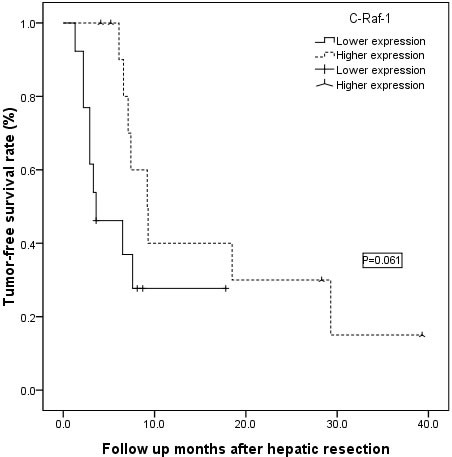
The patients with high levels of c-Raf-1 expression showed similar tumor-free survival rates compared with patients with low levels of c-Raf-1 expression (*P* = 0.061)

Using a grading system based on the levels of tumor histological biomarkers, all 25 of the cases were divided into two groups: a low-expression (0–3) group or a high-expression (4–7) group using the four markers VEGFR-2/3, PDGFR-β and c-Raf-1 (Figure 2). The overall survival rate showed no significant difference between the low- and high-expression VEGFR-2/3 and PDGFR-β subgroup analyses (Figure 2A–2C, all *P* > 0.05); however, the high c-Raf-1 expression group showed significantly longer overall survival than the low c-Raf-1 expression group (Figure 2D, *P* = 0.012). The advantage in long-term tumor-free survival in the high c-Raf-1 expression patients disappeared compared with the low c-Raf-1 expression patients (Figure 3, *P* = 0.061).

### Univariate and multivariate analyses

Additional survival analysis was performed (Table 2) that included 21 factors linked to survival: patient age, BMI, underlying liver disease, HBV-DNA, Child-Pugh score, Hb, PLT, creatine, TB, ALB, NLR, total tumor size, largest tumor size, tumor number, AFP level, risk factors, TNM stage, VEGFR-2, VEGFR-3, PDGFR-β, and c-Raf-1. Univariate analysis was performed using the data shown in Table 2, and patient age equal or younger than 60 years and higher levels of c-Raf-1expression were prognostic factors that predicted both poor overall survival and tumor-free survival. Multivariate Cox regression analyses were performed for these two significant factors, and only higher expression of c-Raf-1 was a significant good factor for overall survival (HR: 1.842, 95% CI: 1.211–2.542, *P* = 0.031) and tumor-free survival (HR: 1.319, 95% CI: 1.017–1.543, *P* = 0.046) in HCC patients who underwent radical hepatic resection followed by sorafenib administration.

**Table 2:** Univariate analyses showing factors that contribute to overall survival and tumor-free survival following LR

Variables	N	Overall survival	Tumor-free survival
		*P*-value	*P*-value
Age≤60 (yes/no)	20/5	0.006	0.044
BMI≥28 (yes/no)	7/18	0.634	0.671
Cause of liver diseases (HBV/no)	23/2	0.640	0.505
HBV-DNA (+/−)	14/11	0.696	0.524
Child-Pugh Score (A/B)	23/2	0.319	0.605
Hb≥120 g/L (yes/no)	21/4	0.277	0.322
PLT≥100×10^9^/L (yes/no)	15/10	0.976	0.975
Creatinine ≥ 90 μmol/L (yes/no)	2/23	0.696	0.467
Total bilirubin≥30 μmol/L(yes/no)	2/23	0.613	0.533
ALB level≥40 g/L (yes/no)	17/8	0.639	0.168
NLR≥4 (yes/no)	4/21	0.569	0.317
AFP ≥800 ng/ml (yes/no)	9/16	0.686	0.417
Total tumor number (1/2-multiple)	21/4	0.622	0.407
Total tumordiameter≥6 cm (yes/no)	16/9	0.756	0.777
Largest tumor size≥6 cm (yes/no)	12/13	0.239	0.243
Risk factors(MI/ST/RMLT1/multiple)	10/1/11/3	0.067	0.663
TNM stage (I/II/III)	1/3/21	0.602	0.585
VEGFR-2 (lower/higher)	12/13	0.395	0.956
VEGFR-3 (lower/higher)	12/13	0.539	0.671
PDGFR-β (lower/higher)	16/9	0.370	0.172
c-Raf-1 (lower/higher)	13/12	0.004	0.020

Abbreviations: M: male; F: female; BMI: body mass index; HBV: hepatitis B virus; NLR: neutrophil/lymphocyte ratio; AFP: alpha-fetoprotein; PLT: platelet; Hb: hemoglobin; VEGFR: vascular endothelial growth factor receptor; PDGFR-β: platelet-derived growth factor receptor; MI: microvascular invasion; ST: satellite targets; RMLT1: resection margins less than 1 cm; TNM: tumor node metastasis.

## DISCUSSION

Hepatic resection has been used as the most common acceptable radical therapy for HCC; however, HCC recurrence remains the main problem following HCC resection. It is known that the presence of vascular invasion, satellite nodules, and resection margins less than 1 cm are significant predictive factors for tumor recurrence in HCC patients [20, 21]. Herein, all of the patients had at least one high-risk factor (microvascular invasion, satellite targets, or resection margins less than 1 cm) for early recurrence, so sorafenib was recommended to all of these patients as a postoperative adjuvant therapy. Although some investigators have reported that preoperative therapies such as chemoembolization or chemotherapy do not show clinical benefit in terms of the prevention of relapse [22, 23], Llovet [24] advocated that research efforts should address the use of local or combination therapies in the adjuvant setting following resection. Angiogenesis plays an integral role in the progression of highly vascular malignancies such as HCC. Some key mediators of these pathways include the following: VEGFR, Raf, and PDGFR. These proteins have increasingly become important for the treatment of HCC because they are targeted by multikinase inhibitors [25]. Sorafenib is a multiple kinase inhibitor that specifically acts on VEGFR 2-3, PDGFR-β and Raf kinases (Raf-1) and prolongs survival time in patients with advanced HCC [12]. Sorafenib as an adjuvant therapy after curative resection has failed in a global, phase III, randomized, double-blind placebo-controlled clinical trial. However, the STORM trail showed that, although not all of the included patients benefit from adjuvant Sorafenib after radical resection, some patients did benefit from this combined therapy. Therefore, in the present study, we analyzed our 25 patients and found that the HCC patients with a higher level of c-Raf-1 expression might benefit from combined hepatic resection and adjuvant Sorafenib. This finding might indicate that the utility of Sorafenib is higher in cases that express high levels of c-Raf-1.

C-Raf-1 is thought to mainly have oncogenic potential and has been found to be highly expressed in certain human and animal malignancies, including HCC [26]. Although the overexpression of c-Raf-1 begins early in a preneoplastic lesion, it is predominantly expressed in basophilic tumors [26]. In the present study, all of the cases showed a high risk for HCC recurrence, even after undergoing radical hepatic resection. Accordingly, these patients were recommended to take Sorafenib as adjuvant therapy; 2 cases with a grade 3 AE who could not tolerate this treatment were excluded from our study, but the remaining 25 cases were continued on Sorafenib therapy. When we compared the long-term outcomes, including overall survival and tumor-free survival, between subgroups, we found only that the cases with high levels of c-Raf-1 expression showed higher overall survival. Although cases with higher levels of c-Raf-1 expression showed increased tumor-free survival compared with cases with lower c-Raf-1 expression levels, this difference did not reach statistical significance. Meanwhile, the univariate and multivariate analyses also showed that lower levels of c-Raf-1 expression were a risk factor for lower overall and tumor-free survival. Our findings have some support from animal experiments. Wang et al. [27] reported that sorafenib inhibited tumor growth and prevented metastatic recurrence after resection in nude HCC mice. Feng et al. [28] examined the role of sorafenib in the prevention of HCC recurrence and found that sorafenib suppressed the development of postsurgical intrahepatic recurrence and abdominal metastasis, which led to prolonged postoperative survival of mice in this model [29].

The main limitations of this study were its limited sample size and retrospective nature. However, to overcome these limitations, and based on the result of this present study, we are now performing a large-sample, prospective study. Additionally, the median follow-up time in this report might not have long enough, so we would like to extend the follow-up time to at least 3 years for each case to observe the long-term outcomes.

In conclusion, patients with higher levels of c-Raf-1 expression on histological analysis might benefit from the administration of sorafenib after hepatic resection for cases with a high risk of tumor recurrence.

## MATERIALS AND METHODS

We retrospectively collected HCC cases from Jan 2010 to Dec 2012; all of these inclusive cases accepted radical liver resection and then received Sorafenib as a postoperative adjuvant therapy. The ethical conduct of this study was approved by our departmental review board in agreement with the 1990 Declaration of Helsinki and its subsequent amendments. The diagnosis of HCC was made on the basis of a positive serum AFP level (>400 ng/ml) with positive imaging findings or at least two enhanced imaging techniques (ultrasound, CT or MRI) showing characteristic findings of arterial hypervascularization in all or part of the tumor and hypoattenuation in the portal-venous phase in high-risk patients [16, 17]. The CT or MRI diagnosis of HCC was based on the presence of lesions with different echogenicity—hypoechoic, hyperechoic, isoechoic, or a mixed pattern—compared with the surrounding liver parenchyma. Livers were examined for tumor size and number, histologic differentiation, and the presence of microvascular and perineural invasion on histological examination. The inclusion criteria included the following: age from 18 to 70; resectable HCC; Child-Pugh score of A or B; histological confirmation of HCC with a high risk of recurrence and with MI, STs or RMLT1; and treatment by liver resection and postoperative sorafenib. The exclusion criteria included the following: cardiovascular or cerebrovascular disease that was a surgical contraindication; coagulation disorders; bile duct-derived or mixed liver cancer; having undergone liver transplantation or other radical therapy; or only willing to undergo radical therapy with no postoperative sorafenib. Based on the inclusion and exclusion criteria, 25 HCC cases were included in our present study.

We retrospectively collected data from these 25 cases and analyzed the risk factors that could contribute to tumor recurrence after liver resection and following sorafenib therapy, including baseline demographic characteristics, tumor characteristics, and tumor histological biomarkers—VEGFR-2/3, PDGFR-β and c-Raf-1. The percentages of positive cells were scored into four categories according to staining: 0 for 0%, 1 for 1–25%, 2 for 26–50%, 3 for 51–75%, and 4 for 76–100%. Additionally the staining intensities were also scored into four grades: 0, 1, 2, and 3. The sum of the percentages and intensity scores was used as the final staining score as described previously [18, 19]. Scores were defined as follows: 0–1, negative; 2–3, low expression; 4–5, moderate expression; and 6–7, high expression. Cases were divided into two groups: a low-expression (0–3) group and a high-expression (4–7) group.

All of the surgical resections were conducted by laparotomy with a standard resection of the liver lobe or liver segment using the clamp method. At our center, surgeries are carried out by the chief physician or a deputy chief physician who has more than ten years of surgical experience. The resection uses the clamp method or an ultrasonic knife to implement the standard resection of a liver lobe or liver segment. The resection section was at least 1 cm from the tumor border. Intraoperative *in vivo* radiotherapy and chemotherapy were not applied, and no portal vein chemotherapy was provided. During surgery, we applied B-mode ultrasound at the same time for intra-operative tumor localization and examination after resection.

Sorafenib was administered to the 25 cases beginning two weeks after liver resection. The initial dosage was 400 mg twice daily via mouth. Safety was assessed continuously, and adverse events and serious adverse events (AEs) were graded according to the National Cancer Institute Common Terminology Criteria for Adverse Events version 3.0. The low-dose level included 200 mg twice daily, or withdrawal. Additionally, treatment for AEs, including observation for grade 1 and 2 and dose reduction or withdrawal for grades higher than 2 and then symptomatic treatment, were applied to these cases.

After discharge, follow-up was performed monthly in the 3 months after liver resection, every 2–3 months in the first year, and then every 3–5 months thereafter in the HCC cases. Patients also came to the clinic at our hospital for relevant monthly follow-up examinations, including abdominal ultra-sonography, AFP, liver function testing, and regular blood examinations. Additionally, routine ultra-sonography of the abdomen was performed to check for the recurrence of the tumor every 2–3 months. When the color ultra-sonography indicated a suspicious recurrence, we advised the patients to undergo enhanced CT or MRI inspection in combination with measurement of AFP level. If a recurrence was detected, we recommended the relevant treatment plan (e.g., re-resection, re-RFA, or interventional surgery) to the patients based on their individual situation, the characteristics of the tumor, and other relevant factors. The overall follow-up time was defined as the interval between the first radical therapy and either local tumor progression or the last follow-up.

We used SPSS (SPSS Inc., Chicago, IL, USA) software version 17.0 for the statistical analysis of the data, which were presented as the mean ± standard deviation for normally distributed data. For univariate analysis, we used Student's *t*-test for continuous variables, and the chi-squared test or Fisher's exact test was used to compare categorical variables. The Kaplan–Meier method was used to estimate the overall and tumor-free survival rates. Univariate and multivariate analyses were carried out using the Cox regression method. A P-value <0.05 was considered a statistically significant difference.

## Abbreviations

HCC: Hepatocellular carcinoma
LT: liver transplantation
TACE: transarterial chemoembolization
NLR: Neutrophil/lymphocyte ratio
MRI: magnetic resonance imaging
CT: computed tomography
HBV DNA: hepatitis B virus deoxyribonucleic acid
BMI: body mass index
HBV: hepatitis B virus
HCV: hepatitis C virus
MELD: model for end-stage liver disease
AFP: alpha-fetoprotein
RFA: radiofrequency ablation
M: man
F: female
ALB: albumin
MI: microvascular invasion
STs: satellite targets
RMLT1: resection margins less than 1 cm
TNM: tumor node metastasis
VEGFR: vascular endothelial growth factor receptor
PDGFR-β: platelet-derived growth factor receptor beta.
